# Addressing maternal depression in home visiting: Findings from the home visiting collaborative improvement and innovation network

**DOI:** 10.1371/journal.pone.0230211

**Published:** 2020-04-16

**Authors:** Darius Tandon, Mary Mackrain, Linda Beeber, Nancy Topping-Tailby, Marcy Raska, MaryCatherine Arbour

**Affiliations:** 1 Institute for Public Health and Medicine, Northwestern Feinberg School of Medicine, Chicago, IL, United States of America; 2 Education Development Center, Inc., Waltham, MA, United States of America; 3 School of Nursing, The University of North Carolina at Chapel Hill, Chapel Hill, NC, United States of America; 4 Division of Global Health Equity, Brigham and Women’s Hospital, Boston, MA, United States of America; Ravensburg-Weingarten University of Applied Sciences, GERMANY

## Abstract

**Background:**

Maternal depression is common among low-income women enrolled in home visiting programs, yet there is considerable variability in the extent to which it is identified and addressed. This study examines outcomes related to postpartum depression screening, receipt of evidence-based services, and reductions in depressive symptoms among clients of home visiting programs in the Health Resources and Services Administration’s Maternal, Infant, and Early Childhood Home Visiting Program Home Visiting Collaborative Improvement and Innovation Network (HV CoIIN), the first U.S. national application of the Institute for Healthcare Improvement’s Breakthrough Series (BTS) Model in home visiting programs.

**Methods and findings:**

Fourteen home visiting programs in eight states, serving a geographically and racially diverse caseload of pregnant women and new mothers, took part in the HV CoIIN. Women in participating home visiting programs received the intervention strategies implemented by their program during participation in the collaborative. HV CoIIN strategies included specific policies and protocols for depression screening and home visitor response to screening results; home visitor training and supervision; delivery of prevention and treatment interventions; and tracking systems for screening, referral, and follow-up. HV CoIIN’s proposed primary outcome was that 85% of women who accessed evidence-based services would experience a 25% reduction in depressive symptoms three months after accessing services. Secondary outcomes included an increased percentage of women who were screened for depression within three months of enrollment or birth, who verbally accepted a referral to evidence-based services, and who received one or more evidence-based service contacts. HV CoIIN resulted in improved symptoms among women who accessed services, from 51.1% to 59.9%. HV CoIIN also improved the percent of women screened for depression, from 83.6% to 96.3%, and those with positive depression screens who accessed evidence-based services, from 41.6% to 65.5%. Home visiting programs in this study were early adopters of quality improvement activities, which may limit the generalizability of these results to other home visiting programs.

**Conclusions:**

Home visiting programs can play an important role in closing gaps in maternal depression identification, referrals, service access, and symptom alleviation. Continuous quality improvement and BTS collaborative methods can be used to improve home visiting services in ways that advance national public health priorities and improve population health outcomes.

## Introduction

Maternal depression is a prevalent problem and is especially common in the postpartum period, with 10–22% of all women experiencing major depression after giving birth [1,2]. Low-income families of all races, ethnicities, and geographic locations are disproportionately affected. Among low-income families, 20% experience major depression [3–6], and 35–40% experience depressive symptoms [7,8]. These numbers are double the rates of women of higher socio-economic status, irrespective of race, ethnicity, or geography [9–11].

Postpartum depression potentially creates two generations of suffering, for the mother and for her children, making it a particularly serious and pernicious problem with repercussions that are felt both in the family and beyond. Depressed women tend to be less positive, less spontaneous, and less responsive with their infants [12–14] than women who are not depressed. Postpartum depression has been linked to developmental delays among infants, including difficulties with social interaction, attachment insecurity, and cognitive impairments [15,16]. Research shows that mothers with postpartum depression less frequently use preventive health services and more frequently use ambulatory and in-patient services for illness and injury [17–19]. Both absenteeism and presenteeism (being physically present at work but functioning suboptimally) due to depression have been estimated to result in a loss of $36.6 billion per year in the United States [20,21]. An extensive body of research associates depressive symptoms with many of the same negative maternal and child health outcomes as major depression [22]: diminished capacity for sensitive parenting practices, poorer quality of life, more medical services, and increased mortality rates [22–24].

Maternal depression, including prenatal and postpartum depression, is an important area of focus for federal agencies that provide health and related services to the nation. The mission of the U.S. Department of Health and Human Services (HHS) is to enhance and protect the health and well-being of all Americans [25]. For more than three decades, HHS’s Office of Disease Prevention and Health Promotion has released Healthy People goals: science-based, 10-year national objectives for improving the health of all Americans. Of the more than 1,200 Healthy People objectives for 2020, 34 are focused on maternal, infant, and child health. Healthy People 2020 also includes a developmental objective that focuses on postpartum depression: Maternal, Infant and Child Health Objective 34 states, “Decrease the proportion of women delivering a live birth who experience postpartum depressive symptoms” [26].

Consistent with other federal initiatives focused on the health and well-being of the nation’s diverse populations, the Maternal, Infant, and Early Childhood Home Visiting (MIECHV) Program administered by HHS’s Health Resources and Services Administration (HRSA) is focusing on improving services for pregnant and postpartum women, infants, and young children. The evidence-based MIECHV Program is built on decades of scientific research showing that home visits by a nurse, social worker, early childhood educator, or other trained professional during pregnancy and early childhood improve the lives of women, children, and their families, particularly in the areas of child health and reductions in child maltreatment [27]. In these voluntary programs, trained individuals meet regularly in the homes of at-risk expectant parents or families with young children to provide education and support, with the goal of fostering an array of favorable maternal and child health outcomes. Home visitors evaluate families’ strengths and needs and then provide services that are tailored accordingly [28].

Through HRSA’s MIECHV Program, more than a quarter of a million families have received evidence-based home visiting (HV) services over the course of more than 2 million home visits [29], giving HV the potential to identify and address maternal depression among low-income women in this country. HV programs typically enroll women who are pregnant, with services continuing until their child reaches 2–4 years of age, depending on the HV model. Numerous models currently exist; a recent report highlighted 18 evidence-based models that favorably impact one or more maternal and child health outcomes, use rigorous research designs, and are eligible for MIECHV funding [30]. These evidence-based HV models typically (a) prepare families for childbirth and for the presence of a young child in the home, (b) provide emotional support, (c) discuss infant and child development, and (d) link parents to prenatal and pediatric care. HV models also address psychosocial risk factors (such as maternal depression) that may impede delivery of HV services through in-home strategies or community resources.

Rates of depressive symptoms among low-income women in HV programs are consistently between 45% and 50% [31–34], which are similar to or higher than the rates of depressive symptoms among low-income women not enrolled in HV. In response, HV programs have increasingly integrated depression screening into standard operating procedures, typically incorporating a self-report tool to determine whether a client has elevated symptoms; if elevated symptoms are noted, the client is referred to a clinical supervisor within the program or an off-site mental health clinician for further assessment.

However, while increased screening for postpartum depression is an important step, screening itself is insufficient to meet the mental health needs of women enrolled in HV. Screening generally focuses on identifying women who require treatment for depression, but fails to provide services to women who exhibit depressive symptoms and are at risk for a host of negative maternal and child health outcomes. A growing body of research highlights the importance of integrating into HV programs those interventions that are efficacious in both treating [35–37] and preventing [38,39] postpartum depression. Building on work conducted in other early childhood settings [40], HV programs have employed mental health consultants to build the capacity of home visitors to support the mental health needs of perinatal women and their children [41,42] and to promote practices that enhance depression screening and referral [43].

Because of the high rates of postpartum depression found among HV clients, as well as the significant number of women at risk for postpartum depression, HV model developers (e.g., Healthy Families America) and researchers have identified *addressing postpartum depression* as a critically needed and highly effective enhancement to HV programs [44]—yet, few efforts to incorporate effective interventions and processes across HV programs currently exist. The large and growing number of HV programs nationally that serve perinatal women offers an enormous opportunity to address postpartum depression in a systematic and evidence-based manner [45].

In recognition of this extensive need and untapped opportunity, when HRSA’s Maternal and Child Health Bureau revised the MIECHV performance measurement system in 2016, they included two required measures related to primary caregiver depression. The first focuses on screening for depression, either after the caregiver enrolls in HV services or after birth (for those caregivers who enroll in the HV program prenatally), and the second focuses on referral and receipt of services for caregivers who screen positive for depression. These measures were designed to support strong practices for depression screening among MIECHV-funded HV programs, such as using a validated instrument, and using screening results in a meaningful way (i.e., to connect depressed caregivers to appropriate services and supports).

National performance data on these two measures were first collected in fiscal year (FY) 2017 from MIECHV awardees [46]. On average, nearly 75% of primary caregivers were screened for depression using a validated tool within three months of enrollment in the HV program for caregivers not enrolled prenatally, or within three months of delivery for caregivers who were enrolled prenatally. HV programs employed a variety of validated depression screening tools, with the Edinburgh Postnatal Depression Screen (EPDS) and Patient Health Questionnaire 9-item version (PHQ-9) the most widely used. This average increased more than three percentage points the next year, to 78.5% [28]. These data indicate high and persistent rates of screening for depression by HV programs early in program delivery.

However, in FY 2017, only 38% of caregivers who screened positive for depression and were referred for services by their HV program received one or more service contacts, and this percentage remained relatively unchanged (38.1%) in FY 2018 [46]. This is despite a broad definition of potential referral sites, including perinatal mental health specialists, community mental health providers, and primary care providers. There are many reasons for relatively low rates of service receipt following depression referrals, including difficulty accessing available mental health services due to schedule conflicts, language barriers, differences in cultural values, and negative attitudes toward mental health services.

HRSA’s MIECHV Program funded the Home Visiting Collaborative Improvement and Innovation Network (HV CoIIN) to address the topic of postpartum depression, using the Institute for Healthcare Improvement’s Breakthrough Series Collaborative (BTS) model. This commonly used continuous quality improvement (CQI) model [47] recruits teams of direct-service providers and stakeholders to pursue one shared, specific aim during a defined period of time, typically 9 to 18 months, by applying three core components: learning sessions, action periods, and Plan-Do-Study-Act (PDSA) cycles. BTS has been used successfully to reduce depressive symptoms in clinical settings [48] and to increase enrollment and improve engagement of families in HV [49]. HV CoIIN represents the first test of the BTS model in addressing postpartum depression. It is also the first examination of the BTS model to address postpartum depression in HV programs.

The next sections of this paper describe how the HV CoIIN used the BTS model to address postpartum depression among women enrolled in HV programs, and present results from the HV CoIIN’s efforts to (a) identify women needing services for depression, (b) refer and monitor clients identified as needing depression services, and (c) improve depressive symptoms among women with positive depression screens.

## Methods

Prior to starting HV CoIIN activities, this study was reviewed and approved by Education Development Center’s Institutional Review Board as non-human subjects research for quality improvement. The authors confirm this study is not a clinical trial or direct intervention and was not registered due to non-human subjects research status.

**Fig 1:**
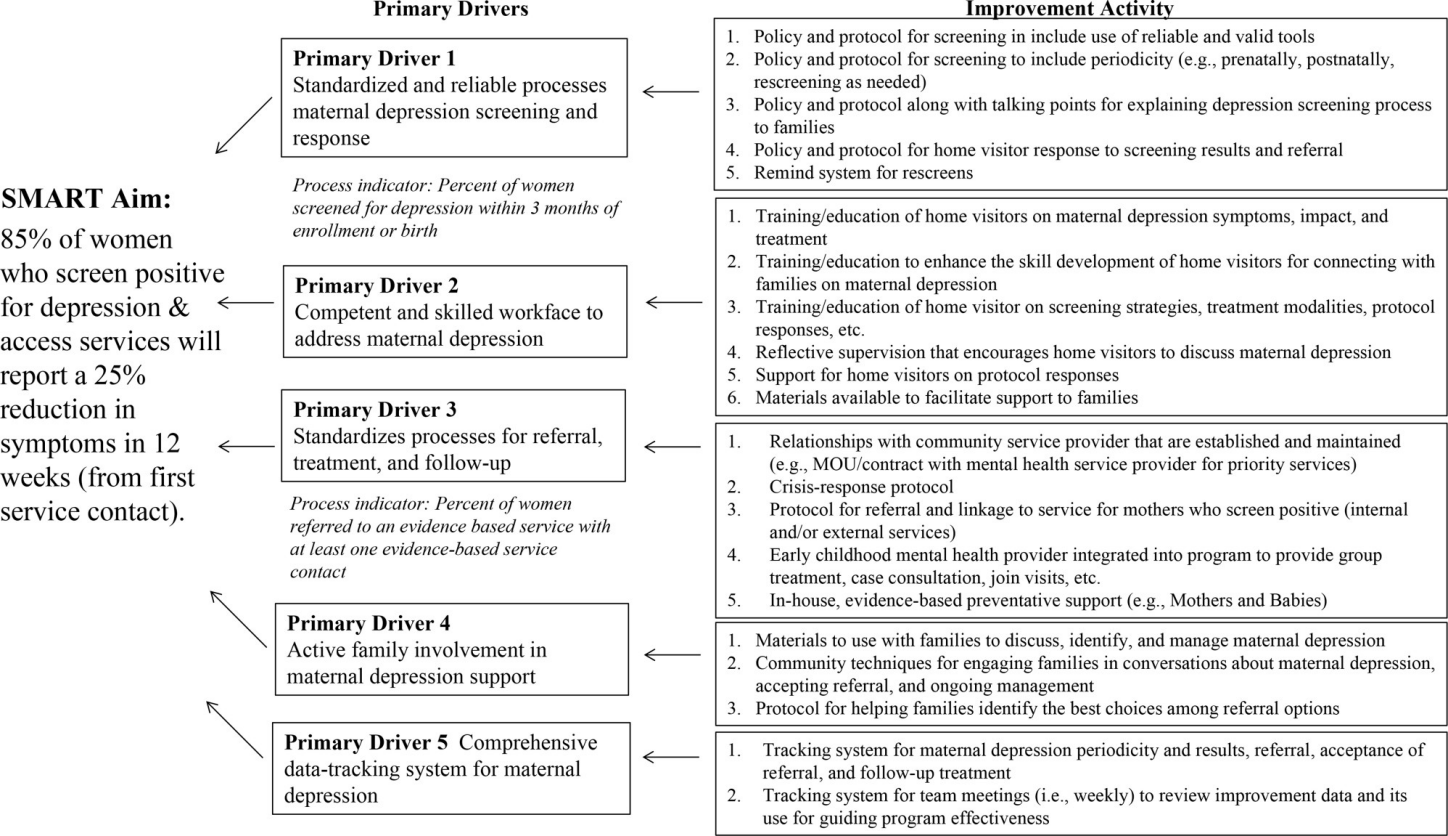
Key driver diagram.

HV CoIIN began by convening a faculty of postpartum depression and CQI experts, HV model developers, and state leaders to develop an operating framework, including (a) a theory of change, (b) a set of outcome measures, and (c) actions for improving postpartum depression screening and referral, access to mental health services, and symptom reduction through HV programs. The HV CoIIN key driver diagram (Fig 1) charted the theory of change in a one-page, visual representation of the HV CoIIN’s specific, measurable, time-bound aim; the elements (primary drivers) that need to be in place for HV programs to reach that aim; and evidence-based changes (improvement activities) that HV programs can use to ensure that those elements are in place and working well. Participating model developers and state leaders helped to align HV CoIIN content with diverse model requirements and state priorities.

### Implementation of the breakthrough series model

HV CoIIN adhered to the three core components of the BTS model: learning sessions, action periods, and PDSA cycles [47]. At the first learning session, the faculty taught the model for improvement, the key driver diagram, measures, and basic CQI skills to members of the local implementing agency (LIA) teams. Together with state representatives, LIAs then signed a charter committing to work together for 15 months to report a common set of measures and to achieve HV CoIIN’s aim.

Central to the BTS model is the use of PDSA cycles in which the evidence-based changes found in the key driver diagram are tested. Each LIA selected one or more evidence-based changes to test during the action periods between learning sessions; teams would also collect data to measure the changes’ effects. LIAs reported on their PDSA cycles and data collection each month to the HV CoIIN. At the second and third learning sessions, teams reported on successes, challenges, and lessons learned in order to share and spread improvements.

The HV CoIIN leadership team provided ongoing support to facilitate collaborative learning. Monthly webinars for LIAs combined (a) faculty instruction on maternal depression, (b) transparent reviews and interpretations of data from the LIAs, highlighting improvements and inviting LIA-based CQI teams to feature their work, and (c) ongoing CQI skill-building (teaching and coaching) from quality improvement advisors. HV CoIIN disseminated to all participants a monthly report that compiled data (time-series graphs of the outcome and process measures, with collaborative-wide averages shown side by side with all individual LIA data), written feedback on each team’s PDSA cycle testing, and highlights of the progress made by individual teams on specific measures, linking that progress to the changes they tested. HV CoIIN maintained an e-list and a website for teams to submit data and share resources. Project leadership also maintained ongoing email communication with states and LIA teams; state leader and model developer stakeholders addressed problems that were beyond the purview of LIA teams.

Two important modifications were made to the original BTS model. First, HV CoIIN added ad hoc, individualized coaching throughout the collaborative to LIA teams and state leaders. Thirteen of the 14 LIAs accessed coaching at least once, and most teams used coaching repeatedly (*M* = 1.75, range 1–5). LIAs most frequently requested coaching on how to (a) design and execute PDSA cycles to maximize learning, (b) create clarity in data reporting, and (c) build an effective team. Occasionally, LIAs received individualized coaching on addressing maternal depression. Among state leaders, two received coaching several times, and one received coaching once. State leaders received coaching on how to (a) develop and run a BTS collaborative, (b) deliver their own coaching to LIAs (e.g., providing feedback on PDSA cycles and data quality), and (c) strengthen state-level staff proficiency in CQI methods and tools.

As the original 15-month completion date approached, LIA teams were just beginning to develop their confidence with and capacity for CQI methods, thus precipitating the second modification: extending the collaborative’s duration. HV CoIIN staff, participants, and HRSA partners reconvened with several high-performing LIA CQI teams to refine the key driver diagram and measures. All HV CoIIN participants were invited to continue in an optional nine-month extension in phase 2. Thus, the full HV CoIIN took place over a span of 24 months in two phases, each consisting of three learning sessions and three action periods.

### Participants

From December 1, 2013 through January 15, 2014, HV CoIIN invited all federally funded MIECHV awardees (50 states, the District of Columbia, and 5 territories) to apply. Ten states with 37 LIAs applied to participate in the HV CoIIN. The LIAs providing HV services included public health departments, intermediate school districts, and nonprofit agencies. LIAs formed internal CQI teams of HV administrators, supervisors, home visitors, and families.

States and LIAs were selected for their capacity and enthusiasm for improvement work. States were required to have an organizational sponsor, staff stability, a CQI leader with time to allocate to CoIIN efforts, data management capacity, a mission for improvement, and the ability to identify three to five LIA partners. LIAs were required to have a senior leader sponsor; a CQI team that included an agency lead, a supervisor, home visitors, and clients; the capacity and willingness to participate for 12–15 months; an interest in maternal depression; and adequate data systems.

In total, as seen in Table 1, HV CoIIN enrolled 14 LIAs serving 1,500 families in eight states and using four evidence-based HV models: Healthy Families America, Parents as Teachers, Nurse-Family Partnership, and Healthy Steps. Healthy Families America and Parents as Teachers use paraprofessional home visitors, Nurse-Family Partnership uses nurse home visitors, and Healthy Steps links a child development expert with pediatric primary care providers. Four states and eight LIAs participated in phase 1; six LIAs joined the four states and four of the original LIAs in phase 2.

**Table 1:** List of participating local implementing agencies.

Local Implementing Agency (LIA)	State	LIA Model	Phase 1 Participation	Phase 2 Participation	Average Monthly Enrollment	Target Population
Batesville School District Parents as Teachers	AR	Parents as Teachers	X		92	Pregnant women, birth–11 months, 12–23 months, 24–35 months, 36–47 months, 48+ months
Blackstone Valley Community Action Program	RI	Healthy Families America, Parents as Teachers		X	149	Pregnant women, birth–11 months, 12–23 months, 24–35 months, 36–47 months, 48+ months
Carolina Health Centers, Inc.	SC	Healthy Families America, Healthy Steps, Nurse- Family Partnership	X	X	430	Pregnant women, birth–11 months, 12–23 months, 24–35 months, 36–47 months, 48+ months
Clark County Combined Health District	OH	Healthy Families America		X	196	Pregnant women, birth–11 months, 12–23 months, 24–35 months, 36–47 months, 48+ months
Community Care Alliance	RI	Healthy Families America		X	66	Pregnant women, birth–11 months, 12–23 months, 24–35 months, 36–47 months, 48+ months
Federal Hill House	RI	Parents as Teachers		X	35	Pregnant women, birth–11 months, 12–23 months, 24–35 months, 36–47 months, 48+ months
Healthy Families Georgia—Columbus Consolidated Government	GA	Healthy Families America		X	43	Pregnant women, birth–11 months, 12–23 months, 24–35 months, 36–47 months, 48+ months
Healthy Families Rappahannock Area	VA	Healthy Families America	X		71	Pregnant women, birth–11 months, 12–23 months, 24–35 months, 36–47 months, 48+ months
Ingham County Health Department	MI	Nurse- Family Partnership	X	X	90	Pregnant women, birth–11 months, 12–23 months
Lac Courte Oreilles Tribal Mino Maajisewin Home Visitation Program	WI	Healthy Families America	X	X	29	Pregnant women, birth–11 months, 12–23 months, 24–35 months, 36–47 months, 48+ months
Little River Medical Center	SC	Healthy Steps		X	74	Birth–11 months, 12–23 months, 24–35 months
Newport News Healthy Families Initiative	VA	Healthy Families America, Parents as Teachers	X		36	Pregnant women, birth–11 months, 12–23 months, 24–35 months, 36–47 months, 48+ months
Oakland County Health Division	MI	Nurse- Family Partnership	X	X	104	Pregnant women, birth–11 months, 12–23 months
Stark County Educational Service Center	OH	Healthy Families America	X		163	Pregnant women, birth–11 months, 12–23 months, 24–35 months, 36–47 months, 48+ months

### Defining outcomes

The HV CoIIN was designed to affect one main outcome: 85% of women who screen positive for depression and access evidence-based services will experience a 25% reduction in symptoms three months after the first service contact. These outcomes were discussed and ultimately decided upon through a collaborative process involving HV CoIIN faculty and were informed by existing examples in the learning collaborative literature that used a 25% improvement in depressive symptom scores as an outcome measure [50]. Screening was conducted using one of two evidence-based tools: the Edinburgh Postnatal Depression Scale (EPDS) and the Patient Health Questionnaire (PHQ) 2 and 9. (PHQ-9 was used when PHQ-2 was positive.) “Positive” screens were defined using the cutoff for mild or greater depressive symptoms specific to each tool: ≥ 10 on the EPDS and ≥ 10 on PHQ-9 [51,52]; these cutoffs were established to include women likely exhibiting major depression and subclinical depression (or, as it is sometimes referred to, minor depression) given emerging research indicating that many of the same negative maternal and child health outcomes occur not only among women exhibiting major depression, but also women with subclinical symptoms [14].

The working definition of “access services” required a client to have at least one contact with an evidence-based service provider. “Evidence-based services” were defined as specific techniques and intervention models shown to have positive effects on outcomes through rigorous evaluation—specifically, cognitive-behavioral therapy (CBT), interpersonal psychotherapy, and medication. Two specific CBT-based interventions with evidence of effectiveness with HV families—*Moving Beyond Depression* [35] and the*Mothers and Babies* course [38] were specifically highlighted as appropriate evidence-based services.

Proximal outcomes included increasing the percent of (a) women screened for depression within three months of enrollment or birth, (b) women with a positive screen who verbally accepted a referral to evidence-based services, and (c) women with a positive screen who verbally accepted a referral and had one or more evidence-based service contacts. As an additional process measure, LIA participants reported the percent of team members who used maternal depression CQI data in practice each month.

### Data collection and analysis

LIA teams reported data monthly through the HV CoIIN website, using a template developed and supported by HV CoIIN staff. The template included (a) a data entry sheet for reporting aggregate program-level data for process and outcome measures; (b) automated LIA-level run charts for each measure; (c) blank sample spreadsheets for home visitors and supervisors to use internally for tracking client-level data to facilitate follow-up, supervision, and reporting; (d) an instruction sheet; and, (e) operational definitions.

Each month, HV CoIIN staff checked the data for completeness and quality, and contacted LIAs to correct errors. Staff then calculated collaborative-wide weighted averages for each measure. Monthly run charts (time-series graphs) tracked the main and proximal outcomes. Staff annotated the timing of specific interventions to help interpret whether changes led to improvement. Collaborative-wide and LIA-level run charts were displayed side by side as “small multiples.” The collaborative-wide chart with weighted averages and all LIA-level charts for one measure were featured on a single page.

Data were analyzed using established methods in the quality improvement literature for identifying “special cause variation,” that is, variation that is unlikely due to chance alone, analogous to statistical significance in traditional enumerative statistics. Specifically, the first 10 data points generated the baseline mean. Two probability-based criteria were then applied to identify special-cause variation: a run of 6 or more points in a row above or below the baseline (shift), and 5 consecutive points increasing or decreasing (trend) [53].

We used STATA 14 to perform data analysis and used SQUIRE 2.0 guidelines in the drafting of the manuscript. An institutional review board process was completed, and the project was identified as exempt, as all data were being used for CQI purposes.

### Client involvement

This study actively involved HV program managers, HV model developers, and state-level officials overseeing HV services in developing the key driver diagram. Our study outcomes were developed by our stakeholders to be client-centered, taking into consideration HV families’ perspectives on the relevance and feasibility of the outcomes. For example, the time period for the proximal outcome for screening (i.e., three months after enrollment or after birth) was selected because stakeholders believed that many clients would not readily divulge information on their mental health within the first one or two months of their HV program enrollment as rapport was being developed with their home visitor.

Once enrolled, HV clients were included on LIA-level CQI teams, participated in HV CoIIN activities (webinars, learning sessions, coaching, etc.), and contributed to the conduct of the collaborative. Many clients attended in-person learning sessions, and several presented during webinars and at in-person learning sessions.

## Results

Participation rates in HV CoIIN activities were high throughout the 29-month collaborative. More than 85% of LIAs attended monthly webinars, 91% reported on their PDSA cycles, and 93% submitted data following their PDSA tests. LIA teams conducted more than 220 PDSA cycles. Importantly, CQI teams also used data in practice: on average, 92% of LIA team members used CQI data in practice monthly, and during the last two months of HV CoIIN, 100% of CQI team members used data in practice.

**Fig 2:**
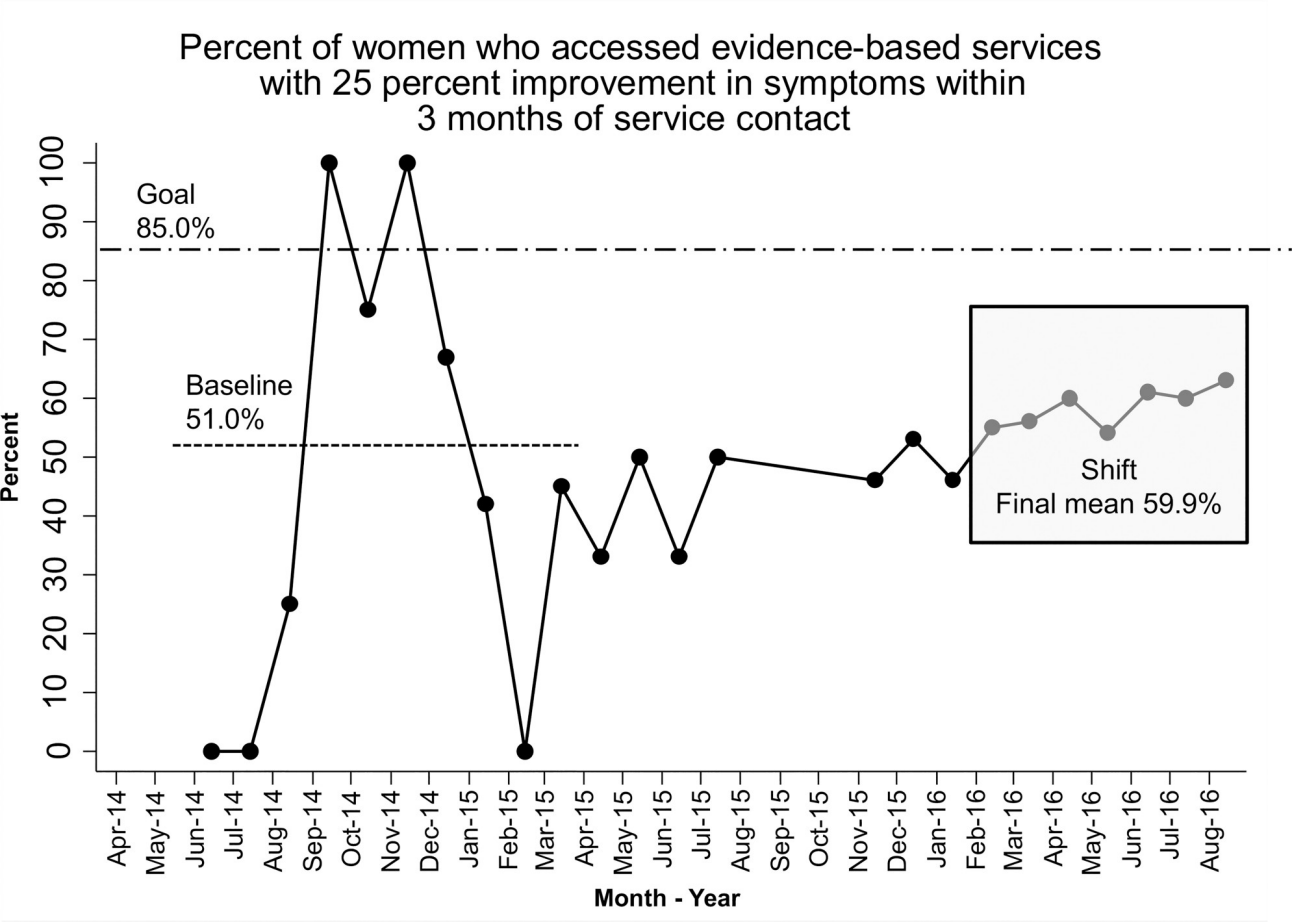
Percent of women who accessed services and saw improvement.

For many LIAs, the expectation that they would report on maternal depression symptom levels on an ongoing basis to HV CoIIN caused them for the first time to re-assess symptoms with a formal re-screen. Initially, the number of women rescreened was small as LIAs developed processes for rescreening, and the percent of women with symptom improvement varied widely from month to month. Baseline data from the first 10 months of the collaborative revealed that an average of 51% of women with a positive screen for maternal depression who accessed evidence-based services experienced a 25% improvement in symptoms within three months of service contact. In this period, 101 women had positive depression screens and were not already receiving evidence-based services, and 42 of them accessed services. By the end of the HV CoIIN, 59.9% of women with depressive symptoms who accessed services experienced an improvement in symptoms within three months of service contact. A shift of 7 points above the baseline signaled improvement (Fig 2); during this period, 84 women had positive depression screens and were not already receiving evidence-based services, and 55 of them accessed services. This shift occurred when a majority of PDSA testing focused on developing (a) in-house capacity to implement non-MIECHV-funded interventions for postpartum depression prevention (e.g., *Mothers and Babies*) and treatment (e.g., *Moving Beyond Depression*); (b) materials and community techniques to engage families in conversations about maternal depression, acting on referrals, and assuming their own treatment management; and, (c) tracking systems (for maternal depression screening periodicity and results, referrals, acceptance of referrals, and treatment follow-up, and for team meetings [i.e., weekly] to review data and their use for guiding program effectiveness).

Earlier PDSA cycles were foundational to these improvements. These cycles focused on policies and protocols for screening, including periodicity, tools, and talking points for explaining depression screening to families; policies and protocols for home visitors’ response to screening results, including crisis response, referral, and linkage to services; home visitors’ knowledge and skills related to maternal depression; reflective supervision to support home visitors in discussing maternal depression; and relationships with community mental health service providers.

**Fig 3:**
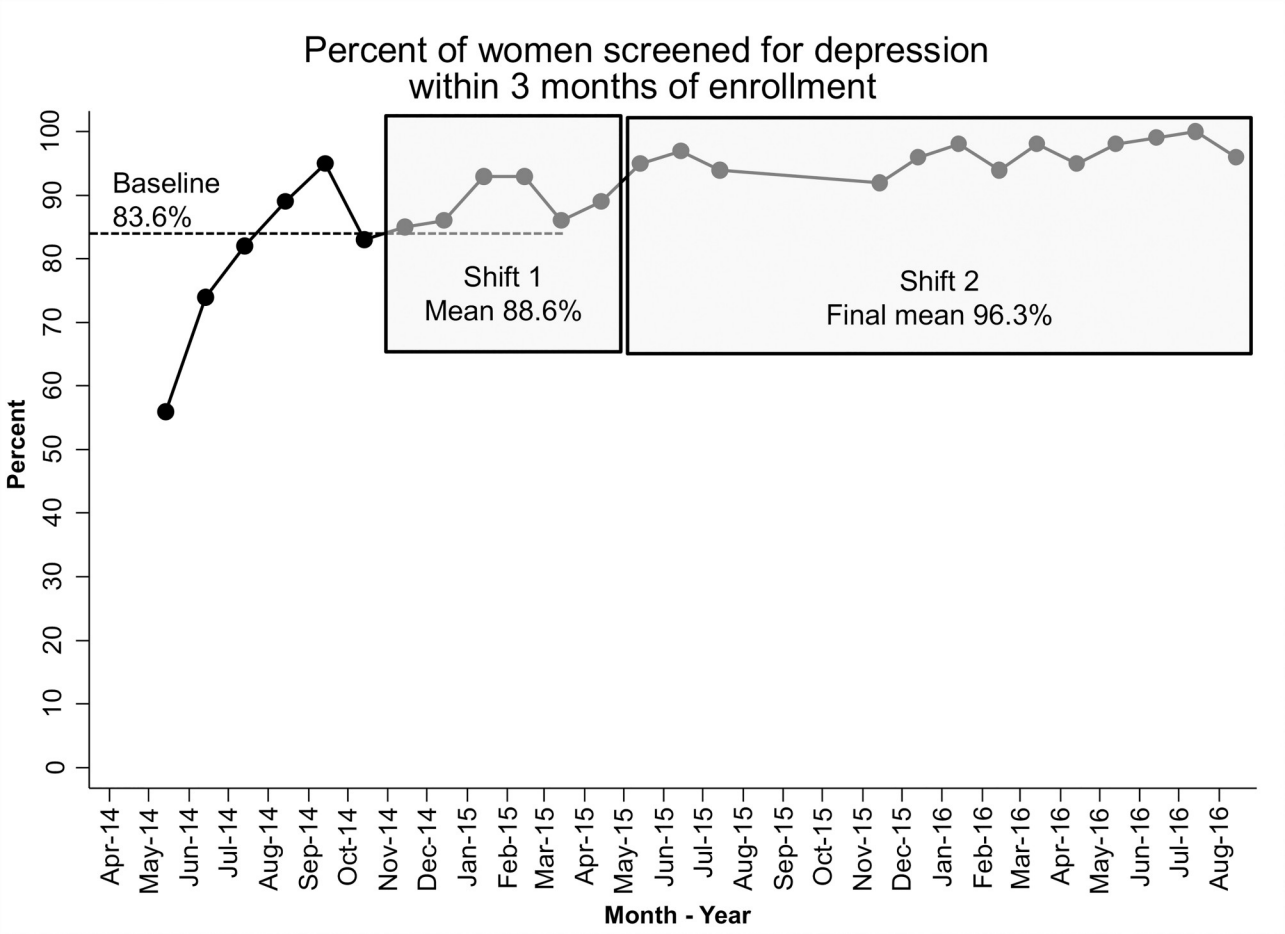
Percent of women screened for depression within three months of enrollment.

Two of three proximal outcomes registered improvement. The percent of women screened for depression within three months of enrollment or birth increased from 83.6% at baseline to 96.3%, with two shifts: one to 88.6% and another to 96.3% (Fig 3). Seven participating LIAs registered improvement on their program-level run charts, and 9 of 14 LIAs (including all who participated in both phases of the HV-CoIIN) met the aim (85%).

At the collaborative level, there was no improvement in the percent of women with a positive screen who verbally accepted a referral to evidence-based services. The verbal acceptance rate was high at baseline (80%) and remained stable and above the goal (85%) throughout the HV CoIIN. Five LIAs showed improvement on their program-level run charts: all five improved from low baseline rates (range 0–70%) to achieve the goal (> 80%).

**Fig 4:**
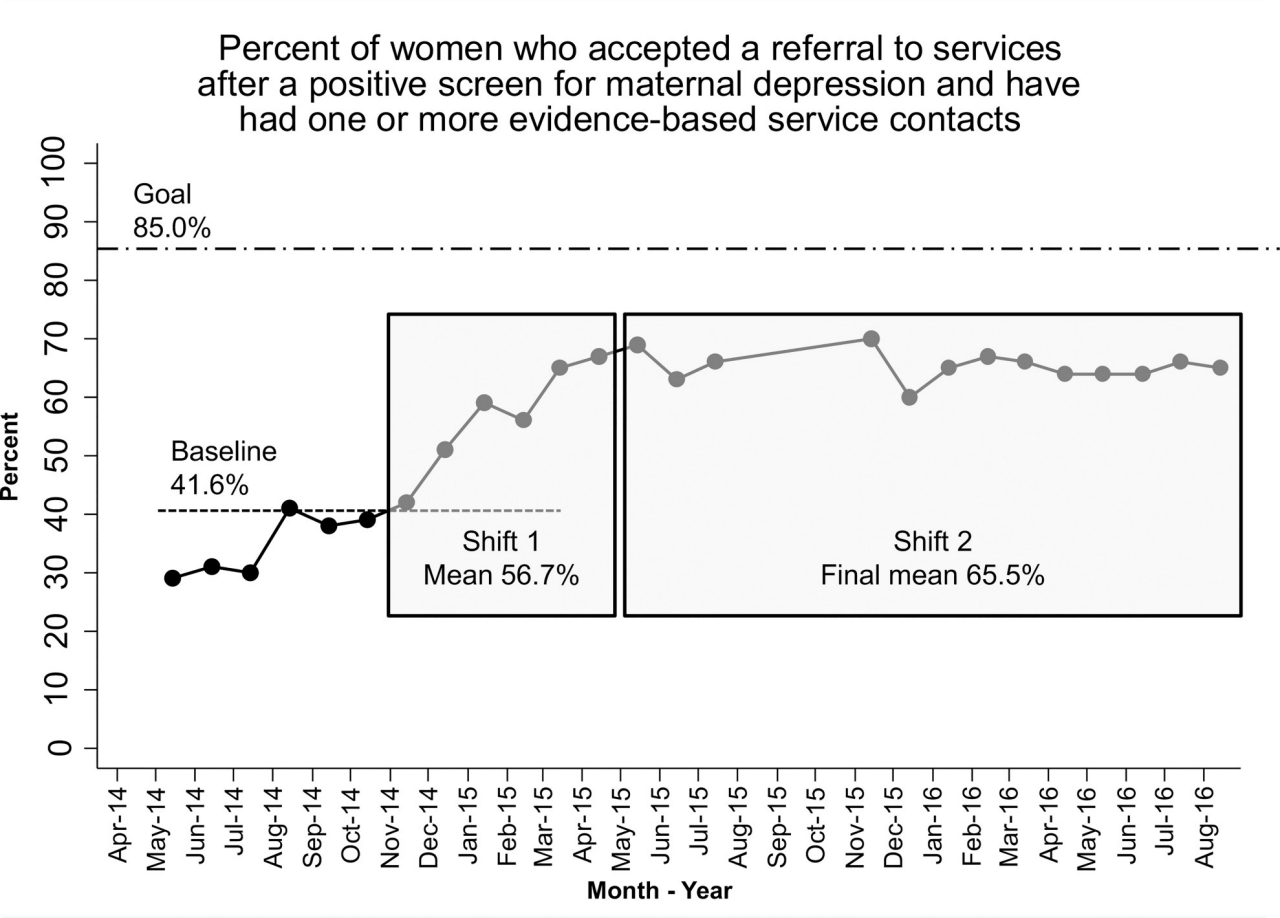
Percent of women who accepted a referral and had an evidence-based service contact.

However, the percent of women with a positive screen who verbally accepted a referral and had one or more evidence-based service contacts increased from 41.6% at baseline to 65.5% by the end of the HV CoIIN. There were two shifts: the first to 56.7% and the second to 65.5% (Fig 4). Six LIAs showed improvement on their program-level run charts, and three met the HV CoIIN aim of 85%.

## Discussion

Using a BTS quality improvement collaborative model with HV programs, HRSA’s HV CoIIN improved rates of depression screening and engagement in evidence-based services, and decreased depressive symptoms among those women who accessed services. Improvement in the main outcome (symptom improvement) coincided with HV programs’ change in focus: from infrastructure and procedural activities (e.g., selecting a standardized depression screening tool or establishing standard operating procedures for screening and referral to evidence-based services) to employing specific evidence-based interventions and services to address symptoms of maternal depression.

HV CoIIN findings are notable given the challenges that HV programs experience in identifying and responding to maternal depression [34,54,55]. Studies also show that maternal depression is a barrier to effective delivery of core HV services [54,56] and that it negatively affects key HV outcomes [57–59]. Considerable additional research shows that HV by itself fails to improve maternal depressive symptoms [44], magnifying the importance of building the capacity of HV programs to address maternal depression through CQI approaches that guide the implementation of evidence-based practices.

This study demonstrated several strengths. Participating sites showed high levels of engagement, with more than 90% reporting data and engaging in PDSA cycles each month, and 100% of team members using CQI data in practice by the end of the study—strong indicators that HV programs were active and consistent in their use of CQI methods to improve recognition of and response to postpartum depression.

This engagement contributed to a second strength: improvements in the proximal and main outcomes. The proximal outcomes—improved rates of screening, referral, and access to treatment—are tightly linked with the standard operating procedures for HV programs. Introducing CQI methods within a collaborative learning network helped teams test adaptations to their existing protocols or supports, learn from one another, and shift from typical practice to best practice. Prior to the HV CoIIN, the main outcome—depression symptom improvement—was considered by most practitioners and stakeholders to be beyond the scope of HV programs.

HV CoIIN’s aim—that 85% of women who access evidence-based services will experience a 25% reduction in depressive symptoms within three months—was ambitious. While its efforts fell short of that goal, the collaborative succeeded in measuring and monitoring this outcome for the first time and also found that nearly 60% of women with postpartum depression experienced reduced symptoms by the end of the HV CoIIN project period. These accomplishments reflect the creativity and vision of the collaborative, and the commitment of the faculty and participating program staff who believed that alleviating women’s symptoms mattered most.

A few limitations warrant mention. HV CoIIN selected early adopter states and LIA participants via an application process, which may limit the general applicability of these results. In addition, with HV CoIIN being extended beyond the originally planned timeframe, and with participation continuing to be voluntary, some participating LIAs changed between phase 1 and phase 2. This change could raise questions of whether some improvements were due to higher-performing LIAs enrolling or lower-performing LIAs leaving the collaborative. None of the signals of change on the run charts, however, coincide with the transition between the two phases. The newly enrolled LIAs in phase 2 entered with baseline data similar to the original cohort, and they made improvements quickly, using previously tested PDSA cycles from the first phase. A final limitation is the possibility of potential sources for confounding, such as simultaneous initiatives external to the HV CoIIN to improve maternal depression screening, referral, and treatment in HV.

## Conclusion

The HRSA HV CoIIN’s effectiveness in improving detection of postpartum depression, receipt of evidence-based services, and symptom reduction shows that learning collaboratives offer a highly effective CQI strategy for HV programs, allowing HV programs to gain expertise in a content area and to engage direct-service staff in learning about CQI processes. The 90% of HV CoIIN’s Learning Collaborative team members who reported using CQI data in practice, and the many phase 1 programs that chose to continue as part of the HV CoIIN, suggest that the processes learned through this collaborative have great potential to help HV programs achieve success in delivering effective, high-quality services that are responsive to their families’ needs.

Additionally, HV programs can play an important role in identifying maternal depression, making successful referrals, and alleviating symptoms. The work of the programs in HV CoIIN demonstrates that CQI and BTS collaborative methods can promote and rapidly spread the effective use of evidence-based practices that in turn can be used to improve HV services in ways that advance national public health priorities and improve population health outcomes. Although the focus of this project’s learning collaborative was on addressing maternal depression, our findings suggest that HV programs—with appropriate guidance from content experts and quality improvement advisors—could engage in learning collaboratives on other health topics that require increased HV attention such as clients’ substance use or trauma histories.

The United States Preventive Services Task Force is a federally supported entity made up of volunteers and national experts, and it remains independent of the federal government and HHS. The Task Force was created in 1984 and is currently authorized by Congress to be implemented by the Agency for Healthcare Quality. It works to improve the health of Americans through the establishment of evidence-based recommendations about clinical preventive services, including screening, counseling services, and medication. Two recent evidence reports from the Task Force highlight postpartum depression; one focuses on the value of screening pregnant and postpartum women for depression to facilitate reduction in depressive symptoms [60], and the other notes a number of effective counseling interventions to prevent postpartum depression [61].

Prenatal and postpartum depression screening received a B rating from a Task force expert panel, suggesting that there is a high degree of certainty that screening of adults, including pregnant and postpartum women, for depression is of benefit and should be implemented within a health care system that ensures accurate diagnosis, effective treatment, and appropriate follow-up [62]. The HV CoIIN facilitated depression screening among HV programs in accordance with the Task Force’s recommendations.

HRSA’s recently funded HV CoIIN 2.0 will work with a new set of HV programs to address postpartum depression, thus providing an opportunity to examine sustained implementation of evidence-based practices among sites included in this study beyond the initial HV CoIIN time period. The interventions tested in HV CoIIN have been bundled into playbooks consisting of a project charter, key driver diagram, a set of PDSA cycles that teams used to get interventions in place and a set of refined measures to help teams monitor implementation efficacy. HV COIIN 2.0 aims to scale the HV CoIIN playbook with up to 25 awardees and an additional 250 LIAs through 2022. Additionally, this playbook is available for public access on the project website. The field of implementation science increasingly calls for studies to examine how evidence-based practices are adapted and sustained over time [63]. Understanding how HV programs adapt interventions with fidelity [64] and what factors they need to sustain evidenced-based practices are key areas for future investigation. Such research may also demonstrate the long-lasting impact of CQI efforts on HV programs’ ability to address such pernicious health issues as postpartum depression.

## Supporting information

S1 Data (DTA)

## Abbreviations

MIECHV: Maternal, Infant, and Early Childhood Home Visiting
HRSA: Health Resources and Services Administration
HV: Home visiting
HV CoIIN: Home Visiting Collaborative Improvement and Innovation Network
LIA: Local Implementing Agency
